# Sensitivity of marine protected area network connectivity to atmospheric variability

**DOI:** 10.1098/rsos.160494

**Published:** 2016-11-16

**Authors:** Alan D. Fox, Lea-Anne Henry, David W. Corne, J. Murray Roberts

**Affiliations:** 1 Centre for Marine Biodiversity and Biotechnology, School of Life Sciences, Heriot-Watt University, Riccarton Campus, Edinburgh EH14 4AS, UK; 2 Department of Computer Science, Heriot-Watt University, Riccarton Campus, Edinburgh EH14 4AS, UK; 3 Center for Marine Science, University of North Carolina Wilmington, 601 S. College Road, Wilmington, NC 28403-5928, USA

**Keywords:** cold-water coral, marine protected area, connectivity, particle tracking, North Atlantic Oscillation, interannual variability

## Abstract

International efforts are underway to establish well-connected systems of marine protected areas (MPAs) covering at least 10% of the ocean by 2020. But the nature and dynamics of ocean ecosystem connectivity are poorly understood, with unresolved effects of climate variability. We used 40-year runs of a particle tracking model to examine the sensitivity of an MPA network for habitat-forming cold-water corals in the northeast Atlantic to changes in larval dispersal driven by atmospheric cycles and larval behaviour. Trajectories of *Lophelia pertusa* larvae were strongly correlated to the North Atlantic Oscillation (NAO), the dominant pattern of interannual atmospheric circulation variability over the northeast Atlantic. Variability in trajectories significantly altered network connectivity and source–sink dynamics, with positive phase NAO conditions producing a well-connected but asymmetrical network connected from west to east. Negative phase NAO produced reduced connectivity, but notably some larvae tracked westward-flowing currents towards coral populations on the mid-Atlantic ridge. Graph theoretical metrics demonstrate critical roles played by seamounts and offshore banks in larval supply and maintaining connectivity across the network. Larval longevity and behaviour mediated dispersal and connectivity, with shorter lived and passive larvae associated with reduced connectivity. We conclude that the existing MPA network is vulnerable to atmospheric-driven changes in ocean circulation.

## 1. Introduction

A global strategic plan was set out by the Convention on Biological Diversity (CBD) in 2010 in response to threats posed by anthropogenic impacts and global change stressors. The plan called for well-connected systems of effectively managed protected areas covering at least 10% of the marine realm is to be set up by 2020 (Aichi Biodiversity Target 11).

Marine protected areas (MPAs) are areas of the ocean that are managed to achieve the long-term conservation of nature with associated ecosystem services and cultural values. In general, individual MPAs do not exist in isolation; these areas are connected in space and time by the movement of migratory species, larval life stages, or simply by the circulation of energy and elements. This means that MPAs form interconnected networks, with the term ‘connectivity’ describing the system of connections in a network.

A core criterion for establishing a well-connected MPA network is that the configuration of the protected areas re-establishes or maintains connectivity of species, habitats, ecological processes and ecosystem services [1]. Global designation of MPAs by CBD signatories is progressing towards this goal [1]. However, even the basic nature and dynamics of connectivity in the oceans are poorly understood [1], with the problem particularly acute in the deep sea, which covers 70% of the Earth's surface (reviewed in [2]). Furthermore, with the world's oceans undergoing natural cycles of change in circulation patterns alongside unprecedented rates of ocean warming, acidification and localized deoxygenation, marine planners must scrutinize the robustness of MPA networks to climate dynamics. Effects of climate change on MPA connectivity are rarely integrated into marine spatial planning [3], yet these changes stand to alter connectivity at timescales directly relevant to management, i.e. from years to decades. In addition to ocean warming, acidification and expanding oxygen minimum zones, the distribution of marine species constantly responds to interannual and multidecadal climate cycles such as the El Niño-Southern Oscillation, the Pacific Decadal Oscillation and the North Atlantic Oscillation. Physical transport of propagules, larval physiology and ecology are affected by changes in the strength and pathway of ocean currents, water temperatures, food supply and mortality rates [4].

In some cases, climate change may increase MPA network connectivity, e.g. warmer sea surface temperatures in the Mediterranean Sea had positive additive effects on larval fish dispersal and adult reproductive timing that would increase network connectivity by 5% over the next century [5]. In other cases, changes in ocean circulation patterns associated with climate change are projected to profoundly affect MPA network connectivity, and mediate effects of ocean warming on larval survival that would have otherwise reduced connectivity [6]. It remains unclear whether such trends can be extrapolated to ecosystems or ocean basins. Nations creating MPA networks to be resilient to climate change must, therefore, strive to gather fundamental information on how populations and sites would be ecologically connected under different climate scenarios [7]. The issue becomes more geopolitically challenging in the case of trans-boundary or High Seas MPA networks because optimal network configuration could change depending on the climate scenario [8]. Our study tackles these issues by analysing the connectivity of a recently established network of offshore MPAs designed to protect vulnerable marine ecosystems formed by cold-water corals.

Cold-water corals can develop elaborate biogenic reef frameworks that are hotspots of biodiversity and vital to global biogeochemical cycles [9]. The vulnerability of these fragile ecosystems to damage by commercial bottom trawling worldwide, their biological importance and socio-economic value prompted international efforts to improve their management and conservation. These ecosystems are now recognized as Vulnerable Marine Ecosystems (VMEs) through the Food and Agriculture Organization of the United Nations (FAO) and constituents of Ecologically or Biologically Significant Marine Areas (EBSAs) through the CBD.

**Figure 1.**
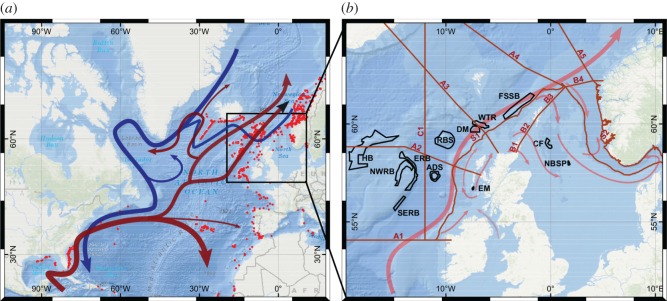
The North Atlantic (*a*) showing the major current pathways and historical observations of *L. pertusa* (UNEP, red dots). The major surface flow through the study area is the North Atlantic Current bringing warm waters northwards. The inset (*b*) shows the study area situated among the densest observations. Also shown are the marine protected areas used in this study (black outlines, key to labels in table 1) and the sections (A1–5, B1–4, C1, S1–2) used to test larval trajectory crossings (dark red lines). The local flows are also shown. The dominant flow is the European Slope Current. Weaker flows occur on the shelf west of Scotland (Scottish Shelf Current), into and across the northern North Sea. Weaker intermediate level flows (not shown) into the basin west of Scotland, offshore of the European Slope Current include Labrador seawater from the southwest and Wyville Thomson Ridge overflow water from the northeast.

One of the most widely distributed reef-building coral is the scleractinian *Lophelia pertusa*, found in all but the Southern Ocean. Its prevalence in the North Atlantic where there is an unparalleled history of data collection coupled with the recent designation of coral MPA networks (figure 1) provides a globally unique foundation to investigate the sensitivity of spatially managed areas to large-scale atmospheric-driven changes in ocean circulation. This study is focused on a network of MPAs designated to protect the cold-water coral *L. pertusa* in seas around Scotland which lie at the heart of the northeast Atlantic distribution, between populations in the Porcupine Sea Bight and Bay of Biscay, south of Iceland and along the coast of Norway.

**Table 1.** Marine protected areas used in this study.

site name	abbr.	type	conserved feature*. L. pertusa* status in the site
Anton Dohrn Seamount	ADS	cSAC	biogenic reef. *L. p.* present
Central Fladen	CF	MPA	no known *L. p.* Contains North Sea oil installations
Darwin Mounds	DM	cSAC	biogenic reef. *L. p.* present
East Mingulay	EM	SAC	biogenic reef. *L. p.* present
East Rockall Bank	ERB	cSAC	biogenic reef. *L. p.* present
Faroe-Shetland Sponge Belt	FSSB	MPA	contains but not designated for *L. pertusa*
Hatton Bank	HB	cSAC	biogenic reef. *L. p.* present
Northwest Rockall Bank	NWRB	cSAC	biogenic reef. *L. p.* present
Norwegian Boundary Sediment Plain	NBSP	MPA	no known *L. p.* Contains North Sea oil installations
Rosemary Bank Seamount	RBS	MPA	seamount communities. *L. p.* present
Southeast Rockall Bank	SERB	SAC	Irish waters. Biogenic reefs. *L. p.* present
Wyville Thomson Ridge	WTR	cSAC	biogenic reefs. *L. p.* present

Coral populations are generally maintained by dispersal and recruitment of sexually produced, pelagic larvae. Dispersal is driven by local circulation patterns, mediated by larval behaviour. The distribution of *L. pertusa* colonies in two shelf regions of the northeast Atlantic has been found [10] to be strongly correlated to a narrow range of water density, possibly suggesting limited movement of larvae outside this density range. However, *in vitro* studies of *L. pertusa* larvae suggest they are able to migrate to the surface, crossing pycnoclines, regardless of light levels [11]. This vertical migration allows larvae to drift with both the subsurface, predominantly density-driven, currents and in the surface, wind-driven, Ekman layer. Population connectivity could, therefore, be highly sensitive to changing patterns in ocean and atmospheric circulation.

Observations of climate change cycles over Western Europe demonstrate that the North Atlantic Oscillation (NAO) is the dominant pattern of atmospheric circulation variability on annual to decadal timescales. The NAO refers to variations in the atmospheric sea-level pressure difference between the Arctic and the subtropical Atlantic [12]. A low-pressure system over Iceland (the Icelandic Low) and a high-pressure system over the Azores (the Azores High) control the direction and strength of westerly winds into Europe. A large difference in the pressure at the two stations (a high index year, NAO+) leads to increased westerlies. By contrast, if the index is low (NAO−), westerlies are suppressed. The strong westerlies during the positive phase NAO bring warm mild conditions to Western Europe alongside a zonal extension of the subpolar North Atlantic gyre which results in larger volumes of warm saline North Atlantic Current flowing in the surface layers off Western Europe [13]. The general trend towards the positive NAO phase from the mid-1990s to mid-2000s appears to be driven by changes in the speed of the North Atlantic jet stream and strength of associated storms, but with significant interannual variability [14]. Correlations between the NAO and current strengths and pathways [13–17] suggest that MPA network connectivity will be sensitive to variations in climate state.

Here we describe how temperature and velocity output from a hydrodynamic model has been used to drive a Lagrangian particle tracking model with larval behaviour for a 40-year period. We apply the biophysical model across NAO states to evaluate the connectivity and its variability in a national MPA network. We mitigate the risk of extrapolating laboratory to real-world scenarios by using virtual larvae with a range of parametrized behaviours designed to cover the uncertainty in larval behaviour. We applied a graph theoretic approach to investigate MPA network resilience to climate change and we discuss the implications of our findings for national management and future design of MPA networks in the High Seas.

## 2. Material and methods

*Lophelia pertusa* reefs are a listed feature in eight protected areas in Scottish waters. While not a listed protected feature, *L. pertusa* also occurs in the Faroe-Shetland Sponge Belt (OSPAR threatened/declining habitats dataset, 2014) so this area has also been included in our analysis. We also include the Southeast Rockall Bank SAC (Special Area of Conservation) in Irish waters, close to the border with Scottish waters and designated for reefs. In addition, two protected areas in the northern North Sea have been included—Central Fladen and Norwegian Boundary Sediment Plain. These sites include oil or gas installations, and while there are no available data to show whether these sites have been colonized by *L. pertusa*, comparison with known occurrences on North Sea oil and gas installations suggests the likelihood is high. They are included here to be representative of such colonies. These protected areas form the network examined here (figure 1 and table 1). The study region ranges from shelf seas to deep sea below 2000 m, including offshore seamounts and banks. *L. pertusa* populations are found in the region at depths from less than 100 m on oil installations in the North Sea to over 1000 m on Anton Dohrn Seamount.

Adult *L. pertusa* are sedentary, fixed to the sea bed. While populations can spread locally through cloning, wider spread is achieved by dispersal and recruitment of sexually produced, pelagic larvae. This dispersal is driven by the local hydrography, mediated by larval behaviour. *In vitro* studies of *L. pertusa* larvae suggest a dispersal potential of up to 50 m per day, and that until larvae are ready to settle they migrate to the surface, crossing pycnoclines, regardless of light levels [11]. However, extrapolating from laboratory-reared corals to larval behaviour in the real ocean is difficult.

The dominant oceanographic feature of the region (figure 1) is the northward flowing European Slope Current along the continental slope [15,18–21]. Intrusions of the European Slope Current onto the shelf occur in the winter months [21,22], possibly as a result of instabilities in the slope current [23,24]. Considerable interannual variability in the shoreward extent of waters of Atlantic origin has been observed [25]. Offshore, west of the European Slope Current, there is weak anticyclonic recirculation of the upper layers within the Rockall Trough, and anticyclonic circulation around individual seamounts [26,27]. In intermediate layers, a cyclonic gyre in the Rockall Trough is fed by Labrador seawater from the southwest and more saline waters overflowing the Wyville Thomson Ridge [28,29] from the north. Inshore, the Scottish Coastal Current flows northward along the Scottish west coast, with estimates of transit times of 4.5–6 months [30] between the North Channel and Cape Wrath, and nine months between Sellafield and the Pentland Firth [31]. In the northern North Sea, Atlantic water enters through the Pentland Firth, between Orkney and Shetland, and between Shetland and the Norwegian Trench, exiting through the eastern part of the Norwegian Trench. The circulation pattern in the northern North Sea and Skagerrak is mainly cyclonic.

The hydrodynamic model used is the POLCOMS Atlantic Margin Model (AMM). Brief details of the AMM model are given here, more can be found in [32]. AMM covers an area from 19.8333° W to 13° E and 40.11111° N to 64.88864° N. The resolution is 1/9° latitude and 1/6° longitude with 40 s-coordinate levels in the vertical. This results in cell resolutions varying from 7.860 km to 14.177 km in the *x*-direction and 12.348 km in the *y*-direction. AMM was run for 1960 as a spin-up, followed by a 45-year integration for the period 1960–2004. Years 1960 to 1964 are not used in the present study as AMM was continuing to spin up from the initial state.

Forcing was provided by the European Centre for Medium Range Weather Forecasts (ECMWF) 40-year re-analysis (ERA40) meteorological data for the surface. The ERA40 re-analysis ran until September, operational ECMWF data were used to extend the series to 2004. Importantly, these meteorological data include the atmospheric pressure and wind variability associated with NAO. Open ocean boundary conditions were taken from a run of the global ORCA1 application of the Nucleus for a European Model of the Ocean (NEMO) run from 1958 to 2004. Tidal forcing was provided by 15 tidal constituents from a northeast Atlantic tidal model, and tide generating forces were applied across the model domain. The available dataset consists of 25 h means averaged from hourly snapshots, in order to remove tidal effects.

The larval particle tracking model was run off-line using the daily 25 h mean temperature, salinity and velocity fields from the hydrodynamic model. The particle tracking model was written in Python [33] specifically for this study and includes modules for horizontal advection, vertical and horizontal diffusion (Gaussian random walk [34]) and larval behaviour. Full details can be found in the electronic supplementary material.

In each run, virtual larvae were released in the model daily throughout February, the *L. pertusa* spawning time in the northeast Atlantic, over 40 years. Larvae were released from positions selected randomly within each of the MPAs. In each run, each year, 1000 larvae were released from each MPA, which kept the computational load manageable. Tests were run to check the effects of releasing fewer or more larvae (100–100 000). Fewer larvae resulted in significant errors in mean pathways and underestimates of numbers of connections. Above 1000 larvae per MPA, such differences were not significant being an order of magnitude smaller than the interannual variability. Similarly, tests with 1000 larvae per MPA but released from different random positions produced variations much smaller than the interannual variability.

To mitigate the risks involved in transferring laboratory results to the real world, we model three different behaviours, bracketing the laboratory observations. We use the following estimates of larval behaviour as standard [11]. Larvae are initially passive, with maximum vertical mean swimming speed of 0.5 mm s^−1^ developing linearly over the first 14 days. During the first 32 days larvae migrate upwards towards the surface, where they then remain in the upper 20 m. After around 32 days the larvae are considered to have reach competency and begin to descend to the bed. Virtual larvae swimming onto the bed are recorded as being at the bed (for purposes of assessing the ability to settle and connectivity) then reflected back into the model interior. *Lophelia pertusa* inhabit waters in a temperature range of about 4–12°C and larvae outside this temperature range were not considered to be viable. Horizontal larval swimming is ignored, as it is orders of magnitude smaller than either horizontal advection or diffusion. Gaussian random variables (variance 10%) are used to introduce some individual variability around the mean swimming speeds and competency age. The larval lifespan is set to 63 days.

The above behaviours constitute the ‘standard’ run. Two other behaviours were considered. ‘Passive’ behaviour, with much reduced mean swimming speeds (0.01 mm s^−1^); and ‘long-lived’ where competency age and lifespan are doubled (to 64 and 126 days). The hydrodynamic model fields (temperature, salinity, sea surface height and east and northward velocity components) used in this study were daily (25 h) means taken from the 40-year (1965–2004) Atlantic Margin application (AMM) of the POLCOMS model [32,35]. These data are available to the oceanographic community via the BODC (British Oceanographic Data Centre; see Data accesibility).

Two MPAs were considered to be connected if larvae released from one site which have passed competency age and returned to the bed then enter another site. When considering larvae crossing ocean sections, we are interested in larvae which cross the section without returning. Crossing counts were, therefore, of those larvae whose tracks intersected the ocean sections an odd number of times. To check the hypothesis that larval dispersal is linked to the state of the NAO, Pearson's correlation coefficients between numbers of larvae crossing a section each year and the NAO index were calculated. These calculations were all implemented in Python code using matplotlib, shapely and scipy packages.

Mathematical graph theory provides a suite of metrics to understand networks and the roles of nodes within networks. These have been used in analysis of networks of marine protected areas to determine if networks are well connected, to highlight important gaps and stepping stones [36,37] and to look at problems of network design and optimization [38]. In this work, the network being considered consists of just 12 sites, which is easily visualized. Nevertheless, even using conservative assumptions, a network of 12 labelled nodes may be realized in any of at least 2^66^ functionally distinct ways [39], well-chosen metrics enable us to navigate this vast space of potential structures and grasp the distinctive characteristics induced by the experimentally obtained connectivity data. Here we use two common metrics, the set of descendants of a node and betweenness centrality; other possible metrics are discussed in the electronic supplementary material. These metrics are calculated in Python using the NetworkX package. The number of descendants measures the potential extent of gene propagation from a given MPA population, or equivalently, the number of MPA populations that could be re-established from a single given MPA. The measure of betweenness centrality estimates the importance of a population to maintaining connectivity: loss of sites with high betweenness centrality values can rapidly degrade and fragment the network.

## 3. Results

**Figure 2.**
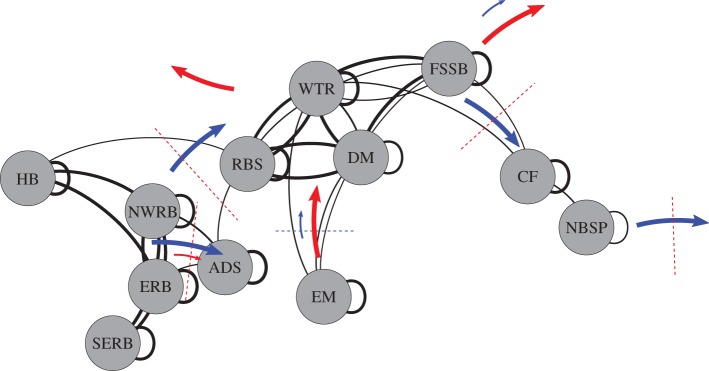
Network connections recorded for the standard run. Lines curve to the right in the direction of the connection and the line thickness shows the strength of the connection. Connections returning to source indicate larvae retained in the site. Site name abbreviations are given in table 1. The western (HB, NWRB, ERB and SERB) and northern (RBS, WTR, DM and FSSB) strongly connected clusters can be seen. Additional arrows and dashed lines show the modelled alterations to the network under positive (blue symbols) and negative (red symbols) NAO conditions. Large arrows indicate strengthened connections and small arrow weakened connections. Dashed lines show connections modelled to be lost. A small arrow and dashed line together shows a seriously weakened connection which would be lost with a larger swing to positive or negative NAO state.

Striking variability in MPA network connectivity was observed, which was strongly correlated to changes in atmospheric circulation. Clusters of strongly connected MPAs were a robust feature of every simulation (figure 2). Two strongly connected clusters of MPAs were identified, in the western extent of the study region (Hatton Bank and the three Rockall Bank sites, henceforth the ‘western’ group) and to the north (Rosemary Bank Seamount, Darwin Mounds, Wyville Thomson Ridge and the Faroe-Shetland, henceforth the ‘northern’ group). A small cluster of MPAs in the North Sea, the inshore East Mingulay MPA and the Anton Dohrn Seamount MPA were only weakly connected to the main clusters. These weaker and less frequent connections between clusters were highly sensitive to NAO state, which changed the fundamental characteristics of the network. Positive NAO state was correlated with a more strongly connected network. While connections between MPAs in the western and northern groups and into the North Sea were lost during negative NAO years, resulting in a highly fragmented network. The negative NAO state was also associated with dispersal pathways from existing MPAs westward into the open Atlantic Ocean.

Key sites were found to be critical to maintaining overall network connectivity. Hatton Bank was important as an upstream larval source for the network. The Rosemary Bank Seamount MPA was crucial to connecting the northern and western clusters, an output that was robust to variation of modelled larval pelagic duration; this highlights the important role of seamount communities in the wider regional ecosystem.

### 3.1. Mediation of dispersal by larval behaviour

**Figure 3.**
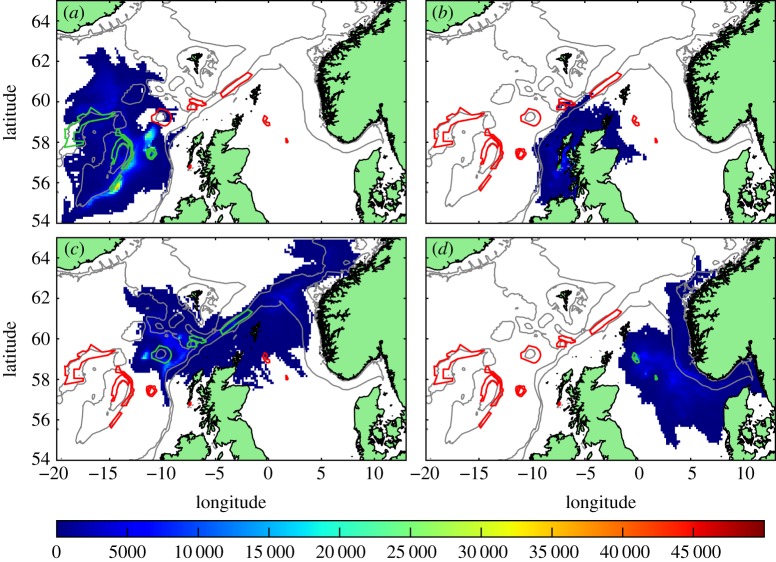
Standard run. Distribution of competent larvae, ready to settle, plotted by source MPAs (shown in green outline, other MPAs red outline). Larval source: (*a*) western MPAs, (*b*) East Mingulay, (*c*) northern MPAs and (*d*) North Sea sites. Colour scale is the number of larval days recorded in the model grid square. Each model day the positions of the larvae are examined and each competent larva in a grid-box counts 1. A figure of 20 000 could, for example, be 20 000 larvae in the grid square for one day each, or 400 larvae for 50 days each, etc.

**Figure 4.**
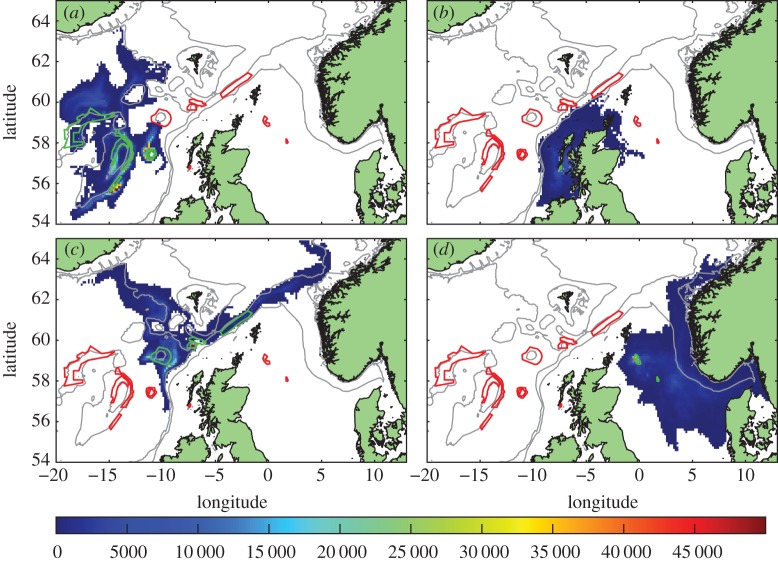
Distribution of competent larvae, ready to settle, in the passive run. Plotted by source MPAs (shown in green outline, other MPAs red outline). Larval source: (*a*) western MPAs, (*b*) East Mingulay, (*c*) northern MPAs and (*d*) North Sea MPAs. Colour scale is the number of larval days recorded in the model grid square.

**Figure 5.**
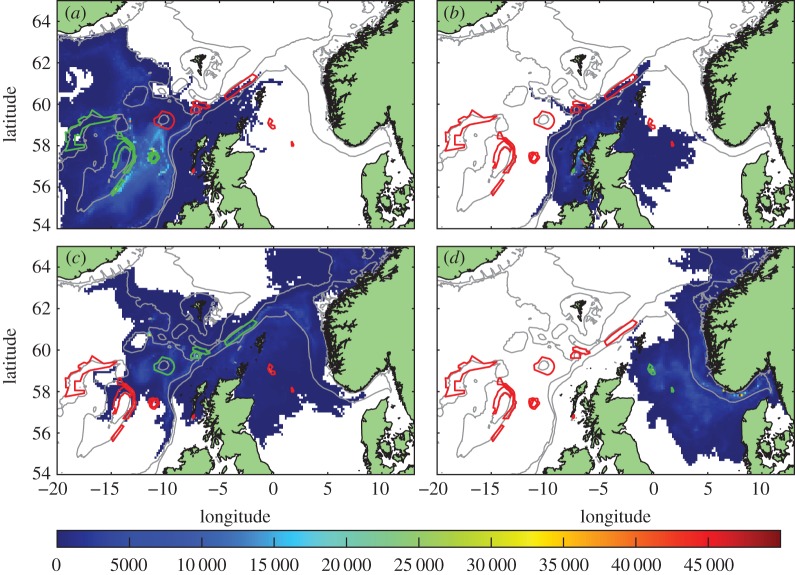
Distribution of competent larvae, ready to settle, in the long-lived run. Plotted by source MPAs (shown in green outline, other MPAs red outline). Larval source: (*a*) western MPAs, (*b*) East Mingulay, (*c*) northern MPAs and (*d*) North Sea MPAs. Colour scale is the number of larval days recorded in the model grid square.

Daily concentrations of larvae which had reached competency, accumulated over the 40 years and mapped onto the model grid (figures 3–5) show dispersal consistent with the known circulation (figure 1). Virtual larvae released from protected *L.pertusa* populations reach the whole of the Scottish seas, across the North Sea to Norway and westward towards Iceland.

Dispersal distances from the western cluster (figure 3*a*) were relatively short, with mean larval distances travelled of 98 km, compared with 190 km, 181 km and 112 km for the northern, North Sea and East Mingulay MPAs, respectively (table 2). No larvae from the western cluster dispersed onto the shelf in either the standard or passive runs, but when larval lifespan was doubled, larvae dispersed across the whole exent of the continental shelf and slope. Larvae from the East Mingulay MPA (figure 3*b*) largely remained on the shelf in all runs, with a small proportion moving into deeper waters in the vicinity of the northern MPA cluster. On the continental shelf, larvae were predominantly transported with the Scottish Coastal Current, with some larvae entering the North Sea. The distances covered by these larvae suggests significantly faster on-shelf currents than previous radioactive tracer-based estimates [30,31].

**Table 2.** Mean and standard deviation of straight-line distance travelled by larvae released from within the main MPA clusters in each of the runs.

	standard run		passive run		long-lived run	
	mean	s.d.	mean	s.d.	mean	s.d.
western cluster plus ADS	97.6	70.2	86.0	63.2	158.0	85.6
East Mingulay	112.1	74.1	103.3	66.7	175.6	118.5
northern cluster	190.0	116.7	247.6	93.6	308.5	187.2
North Sea	180.8	136.8	116.0	126.8	281.6	170.9

Larval dispersal from the North Sea cluster (figure 3*c*) was consistent with regional circulation: initially larvae moved eastwards before joining the Norwegian Coastal Current, either in the Skagerrak or further north in the North Sea, then following the coast northwards out of the North Sea. There were no larvae transported into the southern North Sea in any of the runs in any year. The northern MPA cluster (figure 3*d*) showed the widest dispersal of larvae. Larvae were transported northeastward in the European Slope Current along the shelf break to the Norwegian coast, southeast into the northern North Sea and northwest along the southern slope of the Iceland-Faroes ridge. A few larvae also dispersed south along the continental slope in deeper currents.

**Figure 6.**
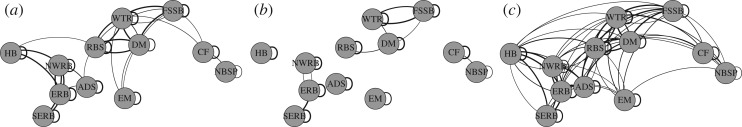
The modelled network connections for (*a*) the standard run, (*b*) the passive run and (*c*) the long-lived run. Connections curve to the right in the direction of larval movement (e.g. all connections at Hatton Bank and East Mingulay are outward). Thicker lines represent connections present in most years, thinner lines infrequent connections.

**Figure 7.**
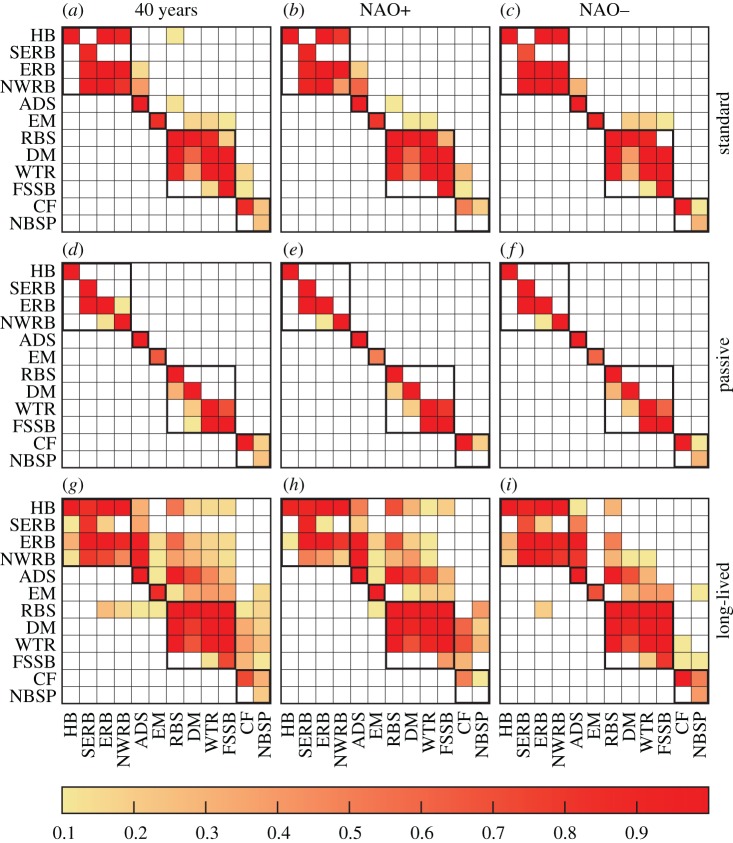
Matrices of larval connectivity between MPAs. Source MPAs are on the *y*-axis, and MPA sinks are on the *x*-axis. Colours represent the fraction of years in which a connection occurred. Isolated MPAs have no connected MPAs in either row or column (e.g. Anton Dohrn Seamount in (*d*)). Pure sources of larvae, with no inward connections, have connections in the row but none in the column (e.g. East Mingulay in (*a*)). Pure sinks, with no outward connections, have connections in the column but none in the row (e.g. Southeast Rockall Bank in (*a*)).

The effects of these different larval behaviours on network connectivity can be seen in figures 6 and 7*a,d,g*. Overall, larvae in the passive run (figure 4) did not travel as far as in the standard run and generally followed the subsurface flows. No cross-shelf-break transport, or connections into the North Sea were predicted by the passive run (figures 6*b* and 7*d*). Anton Dohrn Seamount and the inshore East Mingulay MPA were entirely isolated and no connection was seen between the western and northern clusters. In the long-lived run (figure 5), larvae dispersed more widely and produced many more eastward connections, though the overall network architecture was the same as in the standard run (figures 6*c* and 7*g*) with the same clusters identifyable. The notable exceptions were that longer lifespan enabled larvae from the offshore western cluster to cross the shelf break and reach the inshore East Mingulay MPA; and westward dispersal from Rosemary Bank Seamount was enhanced, reaching MPAs in the western cluster, this potential westward connection was not present in the standard or passive runs.

### 3.2. Barriers and facilitators of larval dispersal

**Figure 8.**
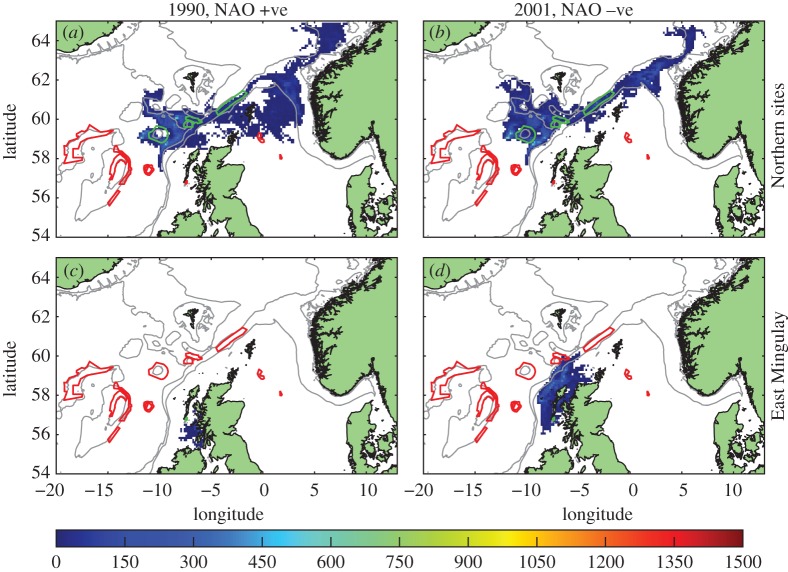
Heat map of competent larval distribution (as for figure 3) for releases in single years, demonstrating interannual variability. (*a*) Source northern MPAs, 1990. (*b*) Source northern MPAs, 2001. (*c*) Source East Mingulay, 1990. (*d*) Source East Mingulay, 2001. Source MPAs outlined in green, other MPAs in red. Scale represents larval days spent in the model grid square.

Larval dispersal and MPA network connectivity and architecture showed high interannual variability (e.g. figure 8). Possible physical drivers of these major interannual network re-configurations, such as effects of wind, dominant currents and topographic features, were investigated by examining larval transport across the ocean sections shown in figure 1. These sections represent physical boundaries (the shelf break, Wyville Thomson Ridge), or cross the main circulation pathways in the northeast Atlantic, features which could act as barriers or facilitators for dispersal and network connectivity.

**Figure 9.**
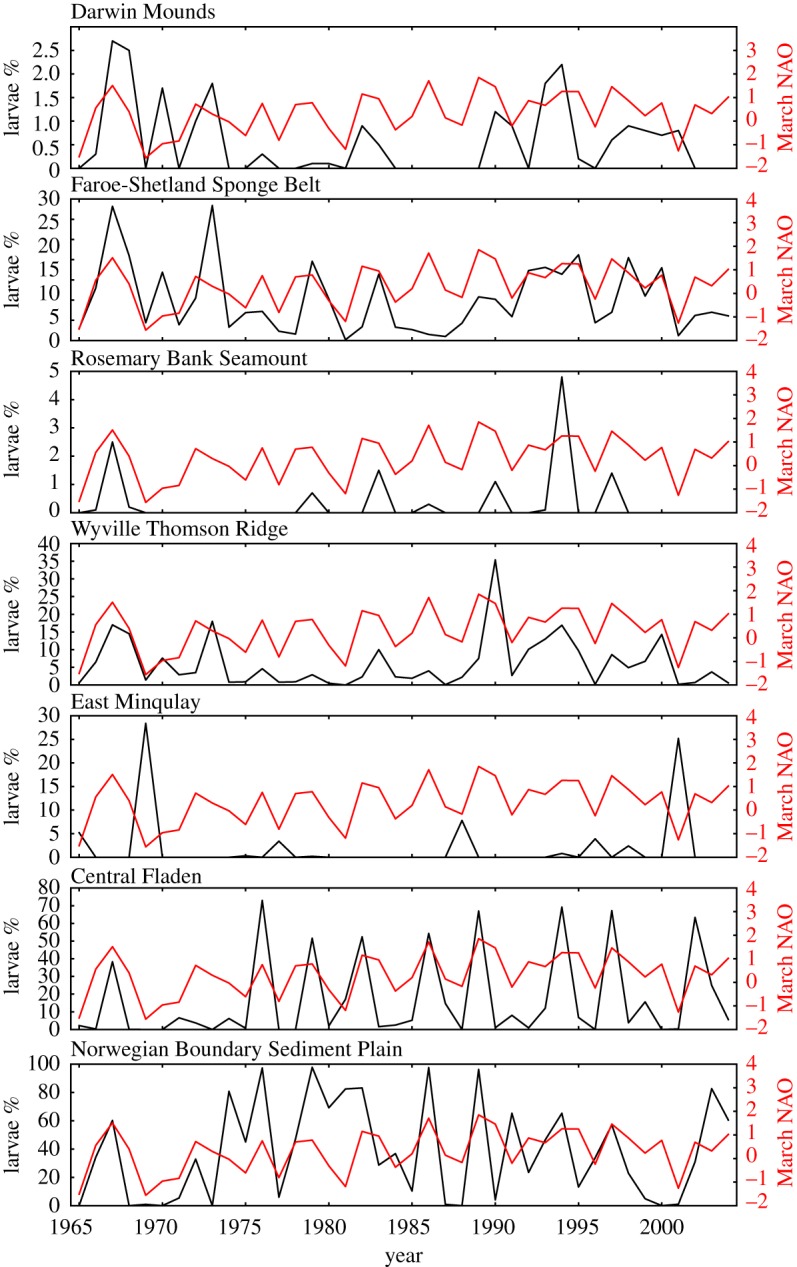
Black line, left scale: percentage of larvae released from each site crossing the shelf break (sections S1, S2) each year. Red line, right scale: NAO monthly average index for March. Source sites East Rockall Bank, Hatton Bank, Northwest Rockall Bank, Southeast Rockall Bank, Anton Dohrn Seamount are not shown as no larvae from these sites cross the shelf break.

Percentages of larvae released from each MPA that crossed the shelf break (200 m contour, sections S1 and S2 in figure 1) in the standard run (figure 9) showed large variability between years (other sections are shown in electronic supplementary material, figures S2–S4). Enhanced cross-shelf larval transport in years of higher positive NAO index was observed for all source MPAs except for the inshore East Mingulay MPA, where reduced transport was seen.

Correlations between the NAO index and larval section crossings for all sections were calculated and are presented in table 3 for the standard run (table 4 for the passive run, table 5 long-lived runs). In the standard run there were significant positive correlations for larvae crossing the shelf break (sections S1 and S2), moving on to the shelf, originating from Rosemary Bank Seamount, Wyville Thomson Ridge and Faroe-Shetland Sponge Belt. By contrast, off-shelf transport of larvae from East Mingulay was significantly negatively correlated with NAO. For larval releases from the North Sea MPAs, transport across the 200 m isobaths into the deeper waters of the Norwegian Trench was significantly positively correlated with NAO. These correlations were consistent with the stronger westerly winds associated with years of higher positive NAO driving the larvae in the near-surface layer towards the European continental shelf, into the northern North Sea and east to Scandinavia.

**Table 3.** Standard run. Correlation between the number of larvae crossing a section annually and the March average NAO index. Numbers in bold indicate *p*-value < 0.01, in italics *p*-value < 0.05. Correlations are not shown when less than 1% of larvae cross a section or when a section passes through the centre of an MPA.

	HB	SERB	ERB	NWRB	ADS	EM	RBS	DM	WTR	FSSB	CF	NBSP
A1												
A2			0.099	0.002	*0.386*	−**0.487**	−0.064		0.044			
A3						−*0.360*	**0.522**	−0.126	−0.294	0.044		
A4								−0.155	−**0.575**	−*0.317*		
A5									−**0.427**	−*0.349*	0.241	0.044
B1						0.127	0.169					
B2						0.014	0.246	*0.339*	*0.363*	−0.126		
B3									**0.530**	**0.536**		
B4									0.170	**0.577**	0.231	0.136
C1	*0.347*	−0.248	**0.646**	**0.549**	−0.249		−0.248	−*0.360*	−*0.388*			
S1						−**0.517**	*0.384*	0.255	**0.497**	**0.505**		
S2									*0.363*	**0.460**	**0.532**	*0.401*

**Table 4.** Passive run. Correlations as for table 3 between the number of larvae crossing a section annually and the March average NAO index. Numbers in bold indicate *p*-value < 0.01, in italics *p*-value < 0.05.

	HB	SERB	ERB	NWRB	ADS	EM	RBS	DM	WTR	FSSB	CF	NBSP
A1												
A2			−0.107	0.275	**0.408**	−**0.601**	−0.254					
A3	−0.281		−*0.374*			−**0.526**		*0.333*	−0.291	−*0.321*		
A4									0.257	*0.349*		
A5									0.214	*0.327*	0.203	0.145
B1						−0.008						
B2						−0.249						
B3												
B4											0.220	0.058
C1	−*0.402*		−0.289		−0.274		0.091	−*0.400*	−0.166	−**0.457**		
S1						−*0.377*						
S2											**0.505**	*0.379*

**Table 5.** Long-lived run. Correlations as for table 3 between the number of larvae crossing a section annually and the March average NAO index. Numbers in bold indicate *p*-value < 0.01, in italics *p*-value < 0.05.

	HB	SERB	ERB	NWRB	ADS	EM	RBS	DM	WTR	FSSB	CF	NBSP
A1	0.197	−0.028	0.126	−0.071	0.155							
A2		−0.100	0.043	0.138	0.023	−*0.343*	−0.027	−*0.379*	−*0.378*			
A3	0.254		0.175	0.195	0.199	−0.227	0.217	**0.494**	−**0.477**	−0.140		
A4							−0.030	−0.019	0.056	0.047	0.038	
A5							−0.130	−0.043	−0.110	0.099	0.188	0.166
B1			0.159	0.159		−0.060	0.239	0.015	0.014			
B2				0.206	0.128	−0.202	0.300	0.104	0.016	−0.106	0.019	
B3					0.128		0.243	0.145	0.291	0.001		
B4							0.008	−0.036	0.047	0.006	0.190	0.124
C1	**0.413**	0.094	**0.466**	**0.590**	−0.107		−0.227	−*0.339*	−0.274			
S1	0.105	0.198	0.311	0.222	0.295	−*0.349*	0.271	0.167	0.158	−0.006	0.019	
S2						−0.017	0.144	0.006	0.254	0.193	*0.324*	0.301

Sections A1–A5 crossed the flows of both the Scottish Coastal Current and European Slope Currents, and tested the correlation of transport parallel to the shelf break with the NAO index. Numbers of larvae from the two seamounts (Anton Dohrn and Rosemark Bank Seamounts) crossing A2 and A3 in a northeasterly direction, respectively, were significantly and positively correlated with the NAO index. By contrast, inshore larval transport from East Mingulay MPA northeastwards along the shelf (sections A2 and A3) was significantly negatively correlated with NAO index. This is a surprising result as a significant positive correlation has previously been reported between the strength of the Scottish Coastal Current through the Tiree Passage and the NAO index in the winter months [15].

Larval transport through the Faroe-Shetland Channel northeastwards along the shelf break towards the Norwegian coast (sections A4 and A5) from Wyville Thomson Ridge and Faroe-Shetland Sponge Belt was also strongly negatively correlated with the NAO index. This contrasted with the passive run (table 4), wherein the corresponding correlations were positive. In the standard run, larvae travelled in the near-surface wind-driven Ekman layer and were driven into the North Sea by the stronger westerly winds in high NAO years, with fewer remaining larvae travelling northeastward through the Faroe-Shetland Channel. In the passive run, the larvae remained in the slope current, below the surface Ekman layer, and transport in this current was positively correlated with NAO in the model. This positive correlation agrees with previous studies in the Faroe-Shetland Channel showing that during positive phases of the NAO the near-surface circulation, below the Ekman layer, tends to be strongly bathymetrically constrained [16], with the negative phase of the NAO associated with a weakened and deflected path of this Shetland slope current.

Sections B1–B4 spanned the northern opening of the North Sea. Larvae entering the North Sea came from the northern cluster, together with a small number from East Mingulay. In the standard run, larval transport into the North Sea from the northern cluster was strongly positively correlated with NAO, with the route larvae took depending on the source MPA. Dispersal pathways from the Darwin Mounds MPA passed between Orkney and Shetland (across B2), while most larvae from the Wyville Thomson Ridge and Faroe-Shetland Sponge Belt MPAs passed to the east of Shetland (B3). In the passive run (table 4), the only route into the North Sea was from East Mingulay. Only a few larvae took this pathway, and correlation with NAO was not significant. In the long-lived run (table 5) transport of larvae into the North Sea was generally positively correlated with NAO index (as for the standard run), but at lower levels of confidence. These results are consistent with studies which show winter-time inflow to the North Sea has a strong connection to the strength in westerly winds, and hence the NAO index [17].

In the standard (table 3) and long-lived runs (table 5), numbers of larvae dispersing eastwards from the western cluster (Hatton Bank, Northwest Rockall Bank and East Rockall Bank) across the north–south section C1 towards the continental slope and shelf were strongly and positively correlated with the NAO index. In the passive run (table 4) where larvae remained in the deeper flows, few larvae crossed C1. Numbers of larvae crossing C1 released from sites to the east of section C1 (Anton Dohrn Seamount, Rosemary Bank Seamount, Darwin Mounds and Wyville Thomson Ridge) were negatively correlated with NAO index, showing stronger westward transport in years of larger negative NAO index. This demonstrates potentially increased supply of larvae westwards into the subpolar gyre and towards the High Seas in conditions of negative NAO.

### 3.3. Network sensitivity to climate state

Having established significant dependence of larval transport pathways on NAO state, the sensitivity of network connectivity and architecture were investigated in more detail by separately calculating connectivity for the 10 years of highest positive and largest negative March NAO years (figure 7). In all three runs (passive, standard, long-lived), connectivity within each MPA cluster was robust to NAO variability. The importance of MPAs as sources of larvae and their roles in keeping the network connected varied with NAO and larval behaviour, but general trends emerged, summarized schematically for the standard run in figure 2.

During negative NAO state, in the standard run (figure 7*a*–*c*), the connection between western and northern clusters was lost, as was the connection into the North Sea. The only connections that strenghtened in low NAO years were those from the inshore East Mingulay MPA to the offshore northern cluster. The connectivity for the long-lived run (figure 7*g*–*i*) showed similar patterns, but links between clusters were weakened rather than lost. But all incoming connections to East Mingulay observed in the long-lived run were lost during low NAO years, while westward dispersal from northern to western clusters was absent during years with high NAO indices.

### 3.4. Critical marine protected area nodes for larval supply and maintaining connectivity

**Figure 10.**
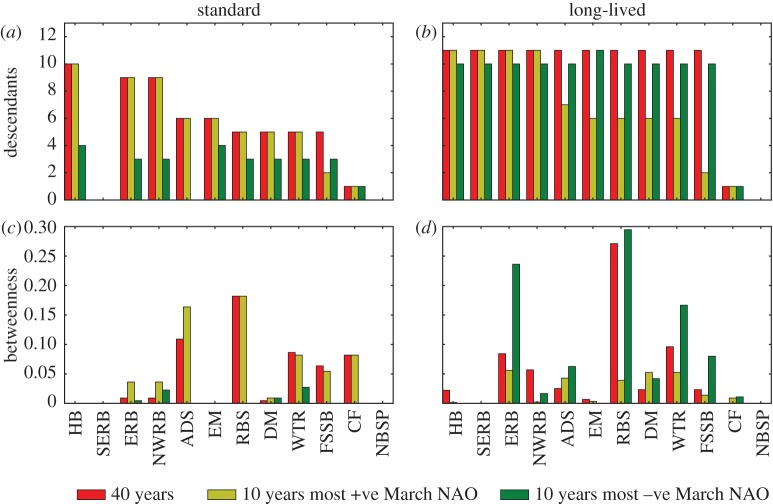
Top row: number of descendants for the network of protected areas produced in the standard run (*a*) and long-lived (*b*) run. Bottom row: betweenness centrality for the standard (*c*) and long-lived (*d*) runs.

Numbers of descendants and betweenness centrality metrics derived from graph theoretics were calculated for each MPA in the standard and long-lived runs for all 40 years, high NAO years and low NAO years (figure 10). The passive run was not shown as the network was almost entirely fragmented.

With regard to the numbers of descendants, the standard run for the full 40-year network and for high NAO years showed the highest values for the western cluster and the Hatton Bank MPA in particular, with the importance of all other clusters decreasing eastwards. Thus, every population except East Mingulay potentially contains descendents of corals from Hatton Bank. By contrast, under negative NAO, MPAs had lower numbers of descendants and the network more fragmented; under this climate state, full population regeneration would require additional sources from MPAs in the northern cluster and from the North Sea. In the long-lived run, the numbers of descendents in the network under negative NAO was similar to the full 40-year network; the biggest change was to the high NAO index years because the network lost the east to west connections. This resulted in decreased numbers of descendants for MPAs in the northern cluster.

Considering the betweenness centrality metric, the standard run for the 40-year network and for positive NAO conditions were again similar, with the most important MPAs being Anton Dohrn and Rosemary Bank Seamounts, which were critical to connecting western and northern MPA clusters. Higher betweenness centrality values for the Wyville Thomson Ridge, Faroe-Shetland Sponge Belt and Central Fladen MPAs were due to their importance in connecting to populations in the North Sea. Low values of betweenness centrality in the network in negative NAO years reflected degradation of network connectivity under this climate state. In the long-lived larvae run, the entire network outside the North Sea is almost fully connected, with each MPA connected to all those east of it. The variability in betweenness centrality reflected the importance of populations to westward connections. Rosemary Bank Seamount was again the most important MPA node, because it provided the only westward link in the network, connecting northern MPAs to those in the western cluster. Low betweenness centrality under high NAO conditions was due to most of the eastward links being single steps (e.g. no MPAs in between) while the westward connections were lost.

## 4. Discussion

Cumulative anthropogenic pressures and uncertainty in the trajectory of change in future ocean conditions add difficulty to the design of protected area networks. However, networks which are designed to be robust under the full range of current variability, rather than just under mean conditions, should be more resilient to future conditions. The widely distributed coral *L. pertusa* spreads locally through sexual and asexual clonal reproduction, so populations in an MPA may continue to thrive for thousands of years but remain isolated from other areas [40,41]. In this context, intermittent connections on the timescales examined here are probably sufficient to maintain genetic connectivity*.* However, a long-term shift in NAO state towards either positive or negative would affect network coherence and site function within the network, particularly if management does not include measures to restrict damage to fragile corals in the MPAs that are most critical to maintaining connectivity and supplying larvae.

The results presented here suggest that management of this national MPA network should assume the worst-case scenario: i.e. that the clusters—western sites, northern sites, North Sea oil and gas sites, East Mingulay and Anton Dohrn Seamount—are not fully interconnected. Similarly, shifts to a positive NAO state will likely isolate populations in the High Seas that are downstream from the national network examined in the present study. A change in mean state of the NAO has been linked to long-term anthropogenic climate change [42], although there remains much uncertainty regarding this link. Thus, adaptive ecosystem-based management plans at the national or international level must account for this worst-case scenario under future states.

Examination of population dynamics and function within the network, via graph theoretic metrics, also provided important pointers for prioritizing protection within the network. The numbers of descendants arriving from the Hatton Bank to other MPAs downstream suggests a critical larval supply role played by populations in this MPA, located in the westernmost reaches of the study region. Another striking result in all scenarios was the critical linking role played by the Rosemary Bank Seamount MPA, which formed a vital stepping stone between the two largest MPA clusters in the west and north according to its betweeness centrality values.

The population represented by the national MPA network seemed intergral to forming downstream connections that crossed geo-political boundaries and even towards the High Seas under scenarios of positive and negative NAO states, respectively. Modelled outputs demonstrated the importance of northern clusters in linking to inshore Scandinavian reefs downstream along the European continental shelf. The only sources of *L. pertusa* larvae to Skagerrak reefs predicted in the model were oil and gas platforms in the North Sea, some of which are extensively colonized by corals. But genetic data [40] show a historical link between the Skagerrak and northeast Atlantic region, suggesting either ocean conditions have changed as the Skagerrak was colonized, or the colonization was an anomalous event not represented in the 40 years modelled in this study. Alternatively, patches of natural reefs may have existed in the North Sea in the past. As North Sea oil and gas installations enter a phase of decommissioning and removal of infrastructure in many instances, effects on network connectivity and ecosystem functioning need to be considered. A programme of ‘rigs-to-reefs', for example, could strengthen connections or re-establish lost connections, and increase network resilience.

The link to the High Seas is very intriguing because it demonstrates the high likelihood that a larger basin-scale network of MPAs on the High Seas that integrate the national MPA network considered in this study will also be sensitive to climate state. New key agreements are being developed at the highest policy levels including the CBD to protect Biodiversity Beyond National Jurisdiction and Marine Genetic Resources, which include networks of High Seas MPAs. Our results from the present study stress the need to safeguard such a network with robust management measures such as bottom fisheries closures in key geographical areas particularly under climate states that degrade connectivity.

**Figure 11.**
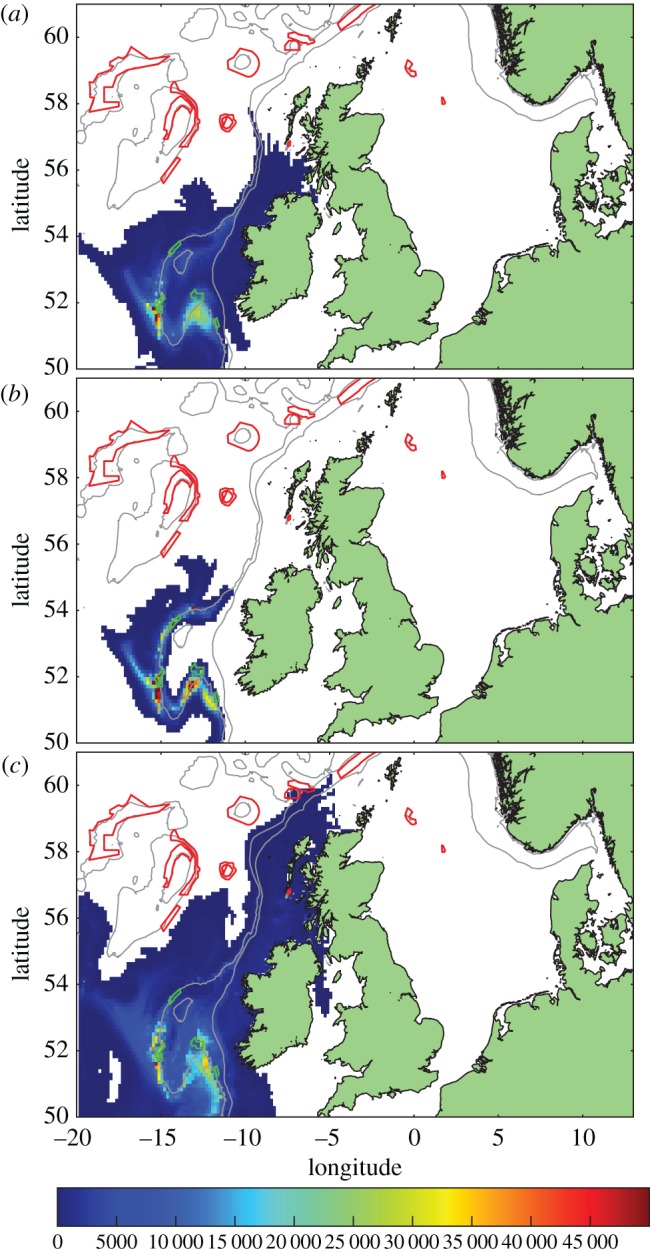
Distribution of competent larvae, ready to settle, released from sites around the Porcupine Seabight. Source MPAs shown in green outline, other MPAs red outline. Model run: (*a*) standard, (*b*) passive and (*c*) long-lived. Colour scale is the number of larval days recorded in the model grid square.

Upstream from this national MPA coral network are well-developed giant coral carbonate reefs in the Porcupine Seabight off Ireland. Modelling larval release from this region (figure 11) suggested the primary link between the Porcupine Seabight and the national network examined in this study may be via the inshore East Mingulay MPA or the northern clusters when larval lifespan was extended.

An additional consideration regards the potential effects of global warming of the oceans on network connectivity. The rate of early development in *L. pertusa* is temperature dependent [43], a rise in temperature from 7–8°C to 11–12°C cutting development time to half, thereby potentially shortening pelagic larval duration. Surface temperature data in the study region examined in this study show mean rates of temperature rise of between 0.3 and 0.5°C per decade since 1985 [44]. Regular subsurface temperature sections [35] show a more complex picture in the surface waters down to 600 m deep, with variability of range about 1.2°C since the 1970s associated with the Atlantic Multidecadal Oscillation and the eastward extension of the North Atlantic subpolar gyre. While it is difficult to extend these temperature data to prediction of future states, the results with different larval lifespans reported here suggest a shift towards a less connected network in a warmer ocean.

Our modelled outputs are consistent with genetic connectivity studies, which show links between the western cluster, the East Mingulay MPA and those in the North Sea [41]. These outputs also support the notion that larvae are capable of significant vertical migration rather than passively remaining within a single watermass or density range [10,45]. Vertical larval migration implies that deep populations may act as refuges to repopulate shallow areas impacted by fishing activities. Predicted transport into the North Sea was weak for larvae with the eight-week lifespan reported by laboratory studies, but much greater for longer-lived larvae. Given the apparent ubiquitous nature of *L. pertusa* growth on oil and gas installations in the northern North Sea [46] this is suggestive of a longer lifespan for larvae in the wild than reported from laboratory observations. This is consistent with newly available results [43] which suggest larvae can survive in the laboratory for up to a year (although in very poor condition and with much of the year after the first two months spent at or close to the bed where currents are weak).

Our study also emphasized the importance of seamounts as links in the connectivity chain, and of the continental shelf slope as habitat, a conveyor belt for along-shore larval transport and a barrier to cross-shelf-break connectivity. However, the physical ocean processes, current variability and cross-slope exchange around both these features are extremely complex [47], thus future work could ascertian the importance of finer-scale hydrographic features such as internal tides or eddies in network connectivity.

In summary, our new findings demonstrated significant climate-driven alterations in the connectivity of an established MPA network using biologically integrative particle tracking models. Dispersal pathways and source–sink dynamics of larvae from habitat-forming cold-water corals were strongly correlated with the dominant pattern of atmospheric variability over Western Europe, the NAO. Wind-driven northward transport of Atlantic waters during the NAO positive phase increased MPA connectivity, whereas clusters of MPAs were isolated during the negative NAO phase and larvae instead tracked a novel pathway eastward into the High Seas. We conclude that the existing MPA network is not robust to atmospheric-driven changes in ocean circulation, and that any future High Seas MPA network in the Atlantic Ocean would also be sensitive to climate dynamics.

## Supplementary Material

A single pdf file, title Supplementary Material, containing more technical description of the particle tracking model and some additional figures. These are not required to support the described results but contain more in depth results and useful information for reproducing the results.

## Data accessibility

This study does not report any primary data. The particle tracking code used to generate the results in this publication is available from the Dryad Digital Repository: http://dx.doi.org/10.5061/dryad.2hf38 [33].

The secondary data used to generate the results presented are:

1. The hydrodynamic model data from the POLCOMS 1964–2004 simulations. These are available from the Natural Environment Research Council's British Oceanographic Data Centre (BODC). These data are NERC data and are made available under conditions in line with the NERC Data Policy. BODC does not routinely assign DOIs to its data holdings, but the data can be found via a request at https://www.bodc.ac.uk/data/online_delivery/numerical_model_data/request/.
2. Marine protected area shapefiles. GIS shapefiles for the Scottish Nature Conservation Marine Protected Areas (ncMPAs) can be downloaded from SNH (Scottish Natural Heritage) Natural Spaces (https://gateway.snh.gov.uk/natural-spaces) and were used under the Public Sector (Scotland) End User Licence, © Crown copyright (and database rights; 2016) OS 100017908 © Crown Copyright, 2015. All rights reserved. Licence No. EK001–20140401. GIS shapefiles for the Special Areas of Conservation (SAC) can be downloaded from SNH Natural Spaces and were used under the Open Government Licence v3.0 (http://www.nationalarchives.gov.uk/doc/open-government-licence/version/3/). GIS files for the SACs in Irish waters can be downloaded from ISDE (Irish Spacial Data Exchange, http://www.isde.ie/) are Copyright Government of Ireland. This copyright material is licensed for re-use under the Creative Commons Attribution 4.0 International licence. http://creativecommons.org/licenses/by/4.0/.
