# Supplementary material for: Sensitivity of marine protected area network connectivity to atmospheric variability
Source: R Soc Open Sci. 2016 Nov 16;3(11):160494. doi: 10.1098/rsos.160494 (PMC5180131; doi:10.1098/rsos.160494)
Supplement: A single pdf file, title Supplementary Material, containing more technical description of the particle tracking model and some additional figures. These are not required to support the described results but contain more in depth results and useful information for reproducing the results. [file rsos160494supp1.pdf]

Supplementary Material

Sensitivity of marine protected area network connectivity to atmospheric variability

Fox et al.

Larval particle tracking model

The larval particle tracking model was run off-line using the daily 25-hour mean temperature, salinity and velocity fields from the hydrodynamic model. The particle tracking model was written in Python and included modules for advection, diffusion and larval behaviour. A simple forward difference numerical scheme with a time step of 1 hour was used throughout.

Advection

Larval position was determined at the start of each time step and the advection step then used the horizontal velocities for the grid-square containing the larva. Comparison test runs were made using velocities interpolated onto the larval position, but differences in the resulting larval distribution and connections between populations were found to be insignificant compared with inter-annual variability and differences due to modelled larval behaviours. A free slip condition was applied for larval movement parallel to the land boundaries, with no motion across land boundaries.

No vertical advection was used. The saved model output does not include vertical velocity fields and the POLCOMS s-coordinate grid, advection scheme and 25-hour averaging makes reconstruction from the continuity condition unreliable, resulting in very noisy derived vertical velocity fields in regions of steep topography. This should not significantly affect results for larvae which can propel themselves vertically with sufficient speed as is the case for *L. pertusa* larvae. However, for passive larvae it may introduce significant errors.

Turbulence and diffusion

The effects of horizontal and vertical turbulence on the larvae were modelled with a random walk[34]:

$$\boldsymbol{x}_{n+1} = \boldsymbol{x}_n + \boldsymbol{K}'(\boldsymbol{x}_n)\Delta \boldsymbol{t} + \boldsymbol{R}\left[\frac{2\boldsymbol{K}(\boldsymbol{x}_n)\Delta \boldsymbol{t}}{\boldsymbol{r}}\right]^{\frac{1}{2}} \quad \textbf{(1)}$$

Where R is a random process with zero mean and variance r. In practice we use a Gaussian random process, G, with $r = 1$. The remaining symbols used are summarised in **Table S1**.

While the AMM uses a turbulence closure model for a spatially and temporally variable vertical diffusivity K_z, these values are not available offline so a uniform diffusivity was used in both horizontal and vertical directions. This approximation is probably only significant in the runs with more passive larvae (smaller vertical swimming speeds, w_p). Here we use $K_x = K_y = 1.0\ \mathrm{m}^2\ \mathrm{s}^{-1}$, $K_z = 0.0002\ \mathrm{m}^2\ \mathrm{s}^{-1}$.

For spatially uniform diffusivity, $\boldsymbol{K}$, and random process G, Equation (1) reduces to:

$$\boldsymbol{x}_{n+1} = \boldsymbol{x}_n + G[2\boldsymbol{K}\Delta t]^{\frac{1}{2}}$$

Larvae which would diffuse out of the surface or onto the bed are reflected back into the interior.

Larval behaviour

The laboratory studies of *L. pertusa* larvae [11] show the larvae are able to swim at speeds up to $0.5\ \mathrm{mm\ s^{-1}}$, with swimming ability developing over the first 14 days of life. The larvae survived for up to 8 weeks and for the first few weeks showed a negative geotactic behaviour, accumulating exclusively in the upper half or one third of the experimental vessels, even when kept in the dark, before later descending to the bottom. Bottom-probing behaviour was evident after between 3 and 5 weeks, but no settlement was recorded in the laboratory. Accumulation of larvae at the water surface in laboratory tanks indicates the ability to move consistently upwards (and later downwards) in the water column. However, the distribution of *L. pertusa* colonies in two shelf regions of the NE Atlantic have been found [10] to be strongly correlated to a narrow range of water density, possibly suggesting limited movement of larvae outside this density range. The average vertical speeds larvae can maintain are difficult to determine from the laboratory observations. A background upward speed of just 0.01 mm s^{-1} superimposed on largely random swimming at up to 5 mm s^{-1} could result in accumulation at the surface in the laboratory tanks 50 cm deep within a few hours. Here we take $0.01\ \mathrm{mm\ s^{-1}} \leq w_p \leq 0.5\ \mathrm{mm\ s^{-1}}$ as the limits of the speed of larval vertical self-propulsion. The lower limit will result in fundamentally passive larvae which do not move far out of the water density range of the parents, the upper limit represents larvae which can move upwards to the surface (and sink down again) at speeds of up to 50 m day^{-1}. The random component of the larval swimming is ignored as it is at least an order of magnitude smaller than the vertical turbulent diffusion. The consistently directed component is determined by:

$$\begin{aligned} 0 < t < T_M: &\quad w_p = f(p, W) \times t/T_M \\ T_M < t < T_{S_p}: &\quad w_p = f(p, W) \\ T_{S_p} < t < T_D: &\quad w_p = -f(p, W) \end{aligned} \qquad (2)$$

Where for particle p, $f(p, W)$ is a Gaussian random function with mean W and variance $(0.1 \times W)$, allowing for some variability in mean swimming speeds between particles. Other symbols are defined in Table S.

This system allows us to vary the behaviour by varying the time to maturity, T_M, the time to start of settling, T_{S_p}, the maximum lifespan, T_D, and the mean vertical swimming speed, W. The settling age, T_{S_p}, is allowed to vary randomly for different larvae, around a central value, T_S:

$$T_{S_p} = R_S \times T_S,$$

where R_S is a Gaussian random variable with mean 1.0 and variance 0.1 T_S.

Vertical swimming speed w_p is set to zero when a particle is within 10 m of the surface. Particles swimming onto the bed are recorded as being at the bed (for purposes of assessing the ability to settle) then reflected back into the model interior.

L. pertusa inhabit waters in a temperature range of about 4–12 °C, larvae were not considered to be viable outside this range. The seas around Scotland are cooler than 12 °C throughout the March–April period being considered. Where modelled larvae entered water colder than 4 °C they died. When selecting random initial positions for larval release, any sites colder than 4 °C were rejected. This happened in two MPAs, Wyville Thomson Ridge and Faroe-Shetland Sponge Belt where the southward flowing, cold deep water is present below about 500 m. This confined larval release in Faroe-Shetland

Sponge Belt to the south east boundary of the site corresponding to the observed *L. pertusa* distribution.

Network analysis, alternative measures of centrality

In the main paper we analyse the network using two graph theoretic metrics: number of descendants, which is relevant to the propagation of genes from a site and network recoverability; and betweenness centrality, which highlights sites which are important for network connectivity. There are many measures of node centrality in graph theory, some more of which are potentially relevant to analysis of ecological networks. Here we briefly present and discuss four of those alternative measures.

In-degree. A simple measure of how many sites a particular MPA receives larvae from. Ecologically, this is probably most useful for highlighting sites with low in-degree, and therefore larvae arriving from a small number of, or no, other sites. These sites could be a source of genetic diversity, but may be difficult to recover if damaged. Identifying such sites could be important for conservation. **Figure S1**(a,b) shows in-degree for the standard and long-lived runs, the pure source sites are clearly seen (East Mingulay and Hatton Bank in the standard run, East Mingulay in low NAO years in the long-lived run).

Out-degree. This is the corresponding measure of how many sites a particular MPA provides larvae to. The relevance to ecological networks is that important sites could be considered to be those which supply larvae to many other sites, making their conservation a priority. **Figure S1** (c,d) shows that all the sites in the main western and northern clusters have similar out-degree in the standard run. In the long-lived run, the upstream (western) sites have increased out-degree, reflecting the important role they play in providing larvae to the network and suggesting the focus of conservation effort in this region.

The following centrality measures can be considered as refinements of in-degree and out-degree, incorporating the importance of sites linked to, or from.

Eigenvector centrality. The fundamental idea is that each node's centrality is the sum of the centrality values of the nodes that it is connected to (or receives connections from), so important sites link to, or are linked to from, other important sites. Left eigenvector assesses links into the site being considered, and right eigenvector assesses links to other sites. This is an attractive idea ecologically, that important sites for conservation are those which supply larvae to other important sites. However, in practice, the relevance of the measure to ecological networks is extremely sensitive to the broad network structure. With networks, such as those discussed here, made up of loosely connected clusters, eigenvector centrality (left and right) focuses all the importance on the sites in the largest cluster (**Figure S1(e,f,g,h)**, standard run). For the more connected long-lived run, left eigenvector suggests more important sites downstream, and right eigenvector more important sites upstream.

Google's *PageRank* algorithm is a refinement of eigenvector centrality. This refinement is primarily for speed and robustness of computation on very large networks, but has important implications for application to ecological networks. **Figure S1**(j,k,l,m) shows that PageRank gives results superficially similar to the corresponding eigenvector centrality. However, the focus on the sites in the largest cluster is reduced. It is interesting to consider how this is achieved. For ease and stability of computation, the PageRank algorithm introduces a complete background network of weak connections (every site connected weakly to every other site). This network has no analogue in the

marine protected area network, making the PageRank results, while superficially attractive, difficult to interpret ecologically.

These measures all provide some insight into the network function. However, no single network theoretic metric produces the importance to ecological network function and conservation of vulnerable sources, isolated sites, stepping stones and well connected sites. This emphasises the need to assess networks against multiple connectivity metrics. None of the metrics discussed include the intrinsic value of larger reefs over smaller reefs.

Supplementary Material Tables

Table S1 List of symbols and abbreviations.

Symbol	Description
$\boldsymbol{x}_{n+1}$	$(x_{n+1}, y_{n+1}, z_{n+1})$, particle position at step $(n+1)$.
$\boldsymbol{x}_n$	(x_n, y_n, z_n), particle position at step n.
$\boldsymbol{K}$	(K_x, K_y, K_z), diffusivity.
$\boldsymbol{K}'$	$\left(\frac{dK_x}{dx}, \frac{dK_y}{dy}, \frac{dK_z}{dz}\right)$,
Δt	Model time step

Supplemantary Material Figures

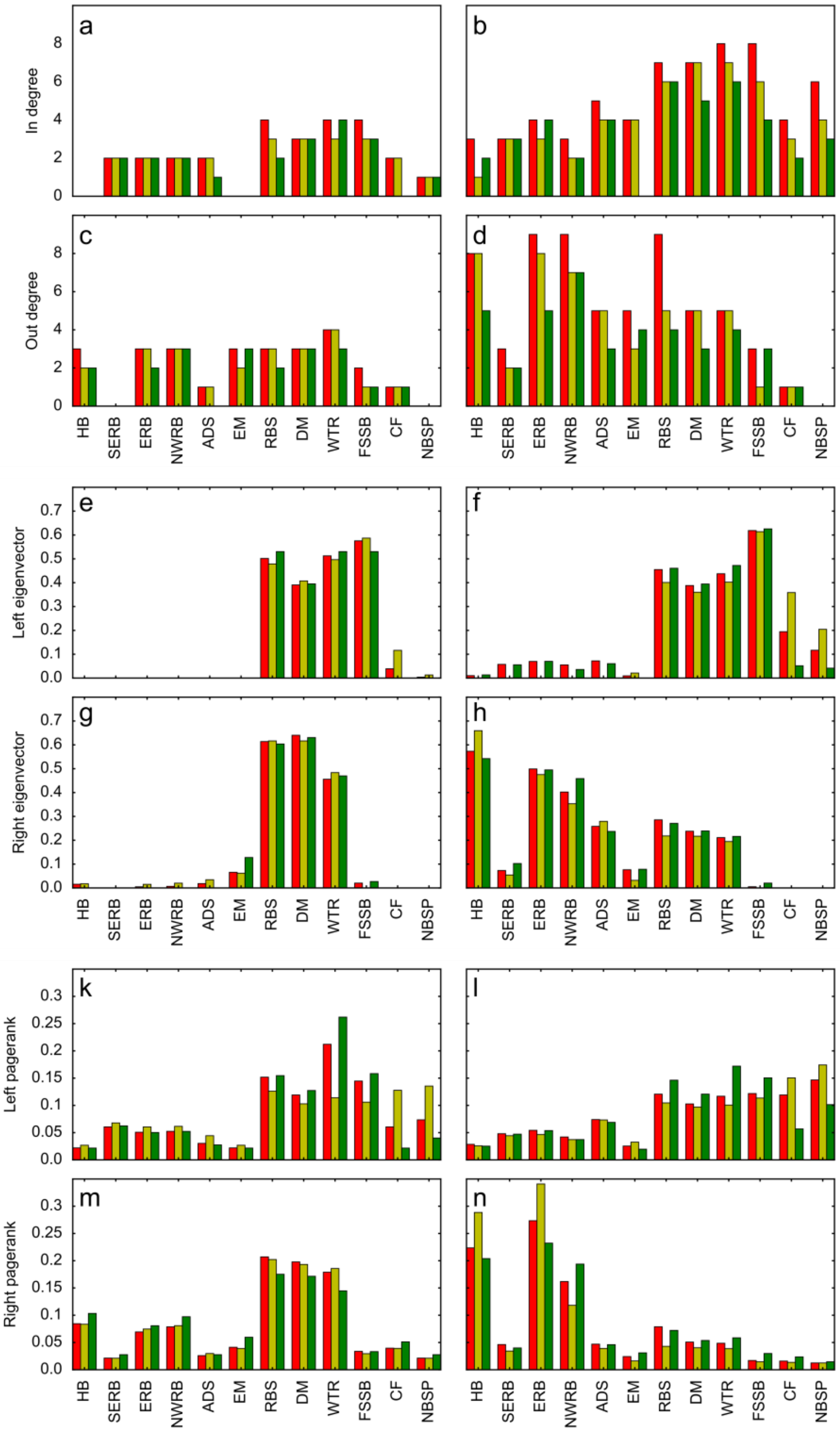

Figure S1 In-degree (a, b), out-degree (c, d), left eigenvector (e, f), right eigenvector (g, h), left PageRank (j, k), and right PageRank (l, m) for the standard (left column) and long-lived (right column) runs. Red bars full 40 year run, yellow bars high positive NAO years, green bars high negative NAO years.

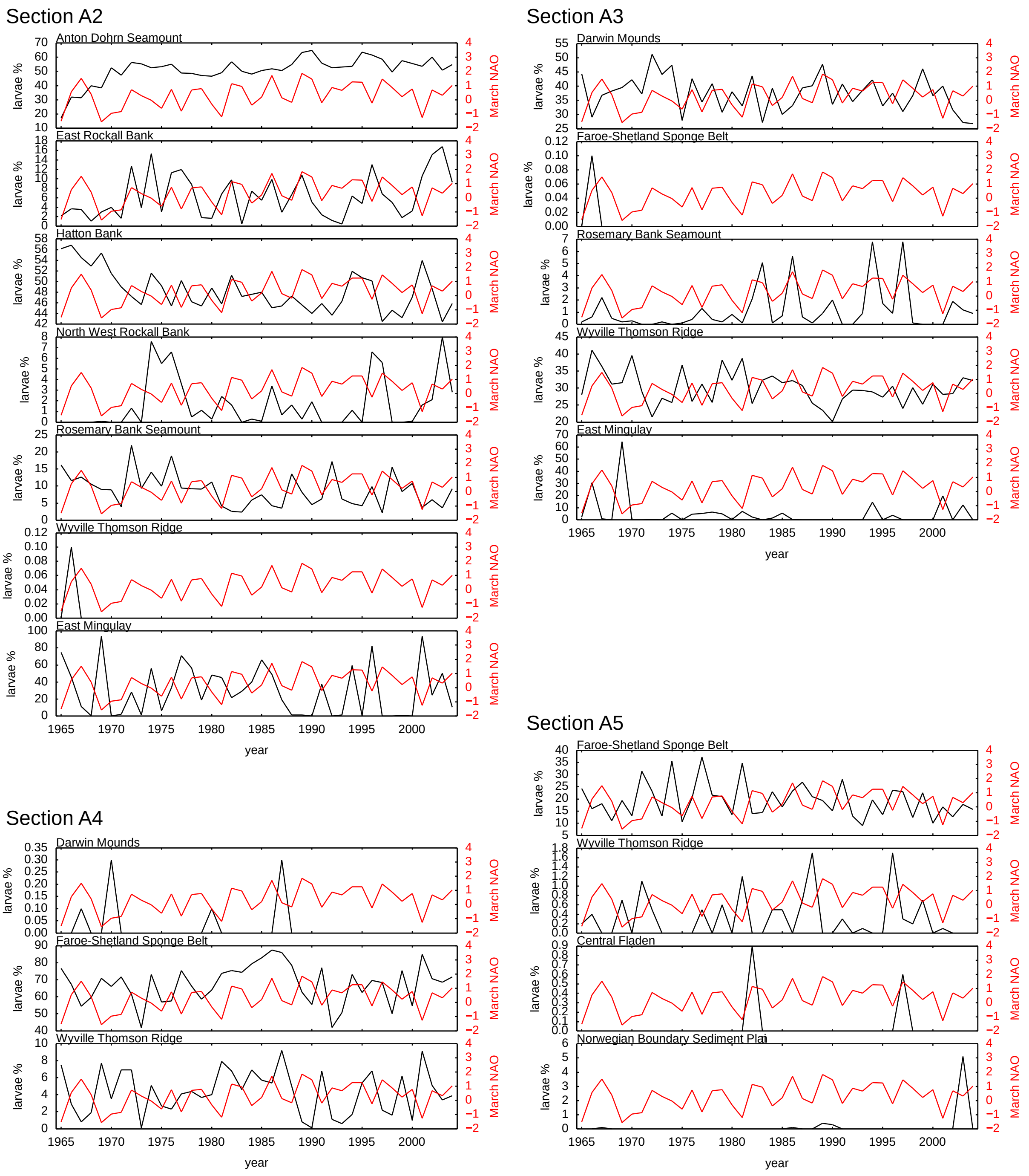

Figure S2 Annual percentage of larvae released crossing section A2, A3, A4 and A5 in the standard run. Black line, left scale: Percentage of larvae released crossing the section each year. Red line, right scale: NAO monthly average index for March. Only source sites with larvae crossing the section are shown. Section A1 is not shown as no modelled larvae cross this section.

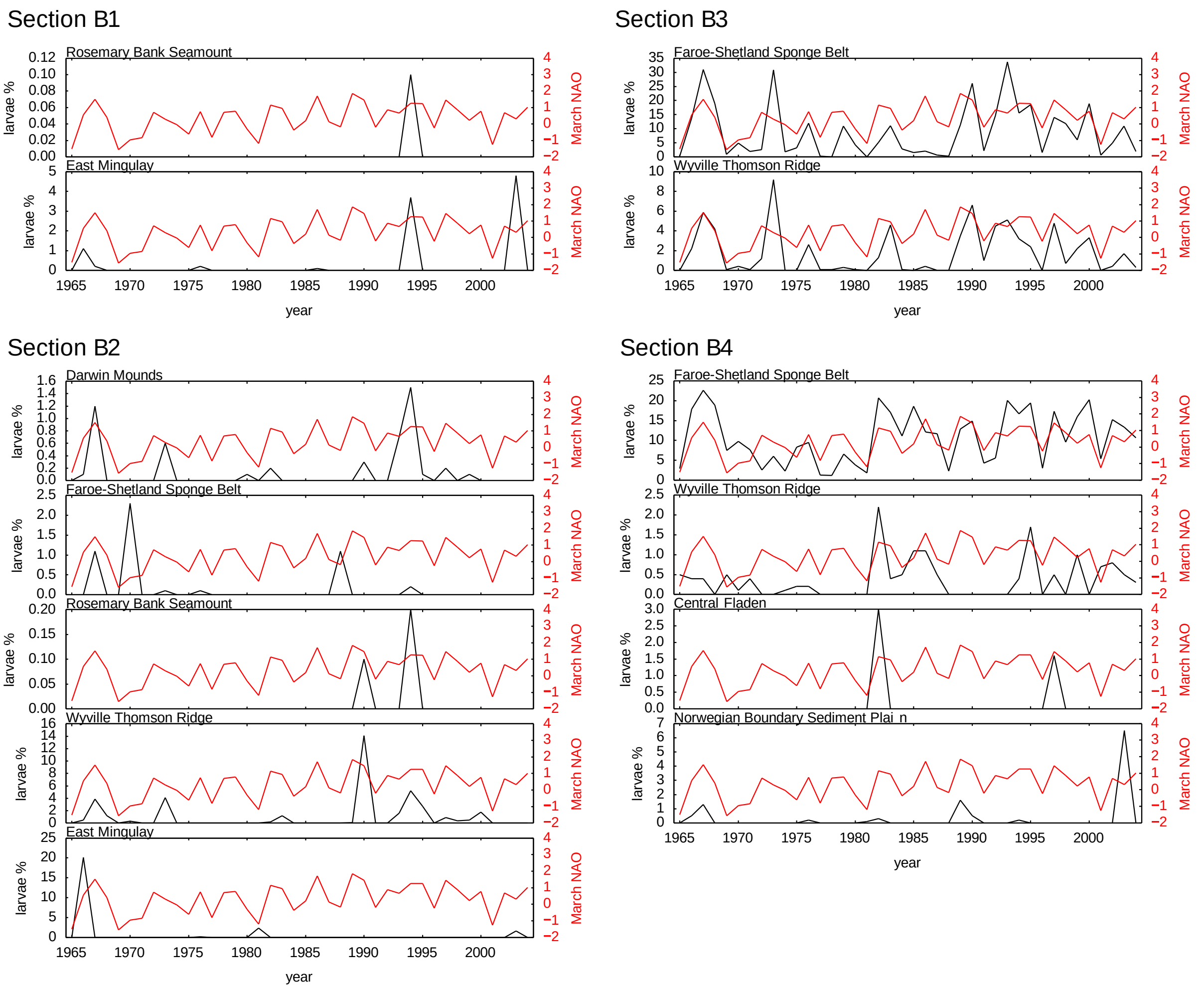

Figure S3 Annual percentage of larvae released crossing section B1, B2, B3 and B4 in the standard run. Black line, left scale: Percentage of larvae released crossing the section each year. Red line, right scale: NAO monthly average index for March. Only source sites with larvae crossing the section are shown.

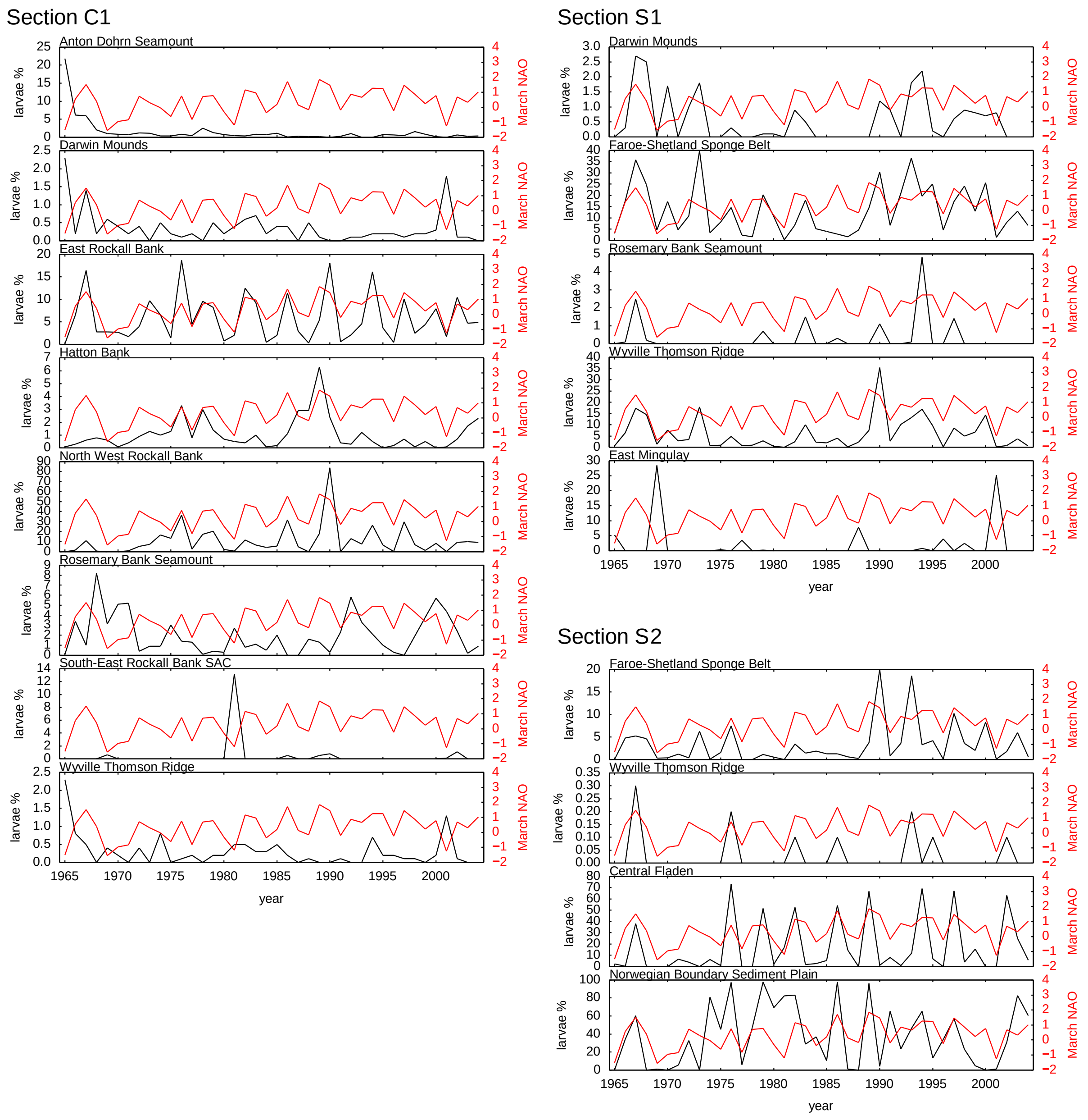

Figure S4 Annual percentage of larvae released crossing section C1, S1 and S2 in the standard run. Black line, left scale: Percentage of larvae released crossing the section each year. Red line, right scale: NAO monthly average index for March. Only source sites with larvae crossing the section are shown.
